# Perturbation theory of a superconducting 0 − *π* impurity quantum phase transition

**DOI:** 10.1038/srep08821

**Published:** 2015-03-06

**Authors:** M. Žonda, V. Pokorný, V. Janiš, T. Novotný

**Affiliations:** 1 Department of Condensed Matter Physics, Faculty of Mathematics and Physics, Charles University in Prague, Ke Karlovu 5, CZ-12116 Praha 2, Czech Republic; 2 Institute of Physics, Academy of Sciences of the Czech Republic, Na Slovance 2, CZ-18221 Praha 8, Czech Republic; 3 Theoretical Physics III, Center for Electronic Correlations and Magnetism, Institute of Physics, University of Augsburg, D-86135 Augsburg, Germany

## Abstract

A single-level quantum dot with Coulomb repulsion attached to two superconducting leads is studied via the perturbation expansion in the interaction strength. We use the Nambu formalism and the standard many-body diagrammatic representation of the impurity Green functions to formulate the Matsubara self-consistent perturbation expansion. We show that at zero temperature second order of the expansion in its *spin-symmetric* version yields a nearly perfect agreement with the numerically exact calculations for the position of the 0 − *π* phase boundary at which the Andreev bound states reach the Fermi energy as well as for the values of single-particle quantities in the 0-phase. We present results for phase diagrams, level occupation, induced local superconducting gap, Josephson current, and energy of the Andreev bound states with the precision surpassing any (semi)analytical approaches employed thus far.

Nanostructures attached to leads with specific properties display interesting and important quantum effects at low temperatures. Much attention, both from experimentalists^1^ and theorists^2^, has been paid in recent years to a quantum dot with well separated energy levels attached to BCS superconductors. In particular, the behavior of the supercurrent (Josephson current) that can flow through the impurity in equilibrium without any external voltage bias between two superconducting leads was in the center of interest^3^,^4^,^5^. The Josephson current through quantum dots with tangible on-dot Coulomb repulsion can induce a transition signalled by the sign reversal of the supercurrent observed experimentally^6^,^7^,^8^,^9^,^10^.

This so called 0 − *π* transition is induced by the underlying impurity quantum phase transition (QPT) related to the crossing of lowest many-body eigenstates of the system from a spin-singlet ground state with positive supercurrent (0-phase) to a spin-doublet state with negative supercurrent (*π*-phase)^11^. In single-particle spectral properties this transition is associated with crossing of the Andreev bound states (ABS) at the Fermi energy as has also been experimentally observed^12^,^13^. Continuous vanishing of the ABS energies at the transition is a direct consequence of crossing of many-body eigenstates^13^ and may serve as an important consistency check of proposed theories. The latter cover by now a broad scope of techniques ranging from numerically exact (and computationally expensive) numerical renormalization group (NRG)^14^,^15^ suitable for zero-temperature and finite-temperature quantum Monte Carlo^16^,^17^ to (semi)analytical methods based on expansion around the atomic limit^18^,^19^,^20^, mean-field theory^21^,^22^,^23^,^24^, or formalisms specialized on the strongly correlated systems such as slave-particles^25^,^26^ and functional renormalization group (fRG)^27^.

However, despite of the versatility of these approaches, there still remain vast regions of the parameter space with direct experimental relevance (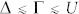, see, e.g., Ref. 8) where most of the above approaches cannot be applied and one has to resort either to overly heavy numerical methods (NRG or QMC) or to conceptually flawed spin-symmetry-broken mean-field approach. The latter approach is not excessively elaborate and often gives quantitatively acceptable results^24^, although at the expense of breaking the spin symmetry of exact solution. In particular, spin-polarized mean-field solutions even after the symmetrization described in Ref. 24 still exhibit at the transition unphysical discontinuities in the ABS energies^24^ and in finite-temperature supercurrents^28^.

The aim of this paper is to provide a conceptually clean and computationally inexpensive generic formalism for addressing the 0 − *π* transition in that widespread regime without strong-correlations (i.e., without the Kondo effect). We show that a resummed perturbation theory (PT) incorporating the second-order dynamical corrections to the spin-symmetric mean-field (Hartree-Fock) solution yields at zero temperature a nearly perfect description of the 0-phase including the position of the phase boundary in a wide parameter range outside of strong correlations. The precision of this solution is unprecedented by any so far employed (semi)analytical methods including fRG. On the other hand, the solution developed from the non-interacting limit breaks down at the phase boundary and any perturbative description of the *π*-phase and, consequently, also finite temperatures which mix 0 and *π* solutions, remains elusive. Although the second-order PT has been applied to this problem previously in [Ref. 23, Sec. V] and, especially, in a (otherwise unpublished) part of Meng's master thesis [Ref. 29, Ch. 4], these studies were limited to the particle-hole symmetric case only (in Ref. 23 in just two limits 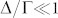 and 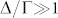) and did not use crossing of the ABS as the definition of the boundary of the 0-phase. Instead, they defined the 0 − *π* transition by equalling the approximated Kondo temperature with the superconducting gap, namely Δ = Γ/(1 − ∂Σ(0)/∂*ω*), which however holds only qualitatively. The generic character of the PT method and the proper definition of the 0 − *π* boundary in the Green-function formalism have thus remained unnoticed.

## Results

A single impurity Anderson model is used to simulate the quantum dot with well-separated energy levels connected to the superconducting leads in the experimental setup^13^,^14^,^16^,^28^. The Hamiltonian of the system consisting of a single impurity with the level energy *ε* and local Coulomb repulsion *U* attached to two superconductors reads
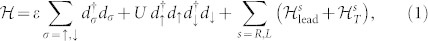
where the BCS Hamiltonian of the leads is
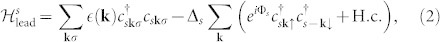
with *s* = *L*, *R* denoting the left/right lead, respectively. Finally, the hybridization term between the impurity and the contacts is given by
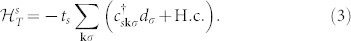


The individual degrees of freedom of the leads are unimportant for the studied problem and are generally integrated out, leaving us with only the active variables and functions on the impurity. Due to the proximity effect there are locally induced superconducting correlations on the impurity and the most direct way to handle them is via the Nambu spinor representation of the local fermionic operators in which the one-electron impurity (imaginary time/Matsubara) Green function (GF) is a 2 × 2 matrix
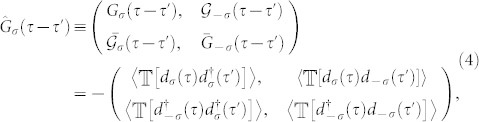
where the bar denotes the hole function.

The impurity GF can be exactly found for an impurity without onsite interaction (*U* = 0) by method analogous to Appendix A of Ref. 19. When assuming identical left and right superconducting gaps Δ*_L_* = Δ*_R_* ≡ Δ as well as tunnel couplings 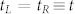 it can be written in terms of Matsubara frequencies *ω_n_* ≡ (2*n* + 1)*π*/*β* as (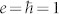 throughout the paper; we also skip the spin index as we only consider spin-symmetric solutions)
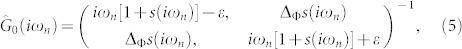
where 
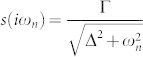
 is the hybridization self-energy due to the coupling of the impurity to the superconducting leads. We have denoted by Γ = 2*πt*^2^*ρ*_0_ the normal-state tunnel coupling magnitude (*ρ*_0_ being the normal-state density of states of lead electrons at the Fermi energy) and Δ_Φ_ ≡ Δ cos(Φ/2) with Φ = Φ*_L_* − Φ*_R_* being the difference between the phases of the left and right superconducting leads.

The impact of the Coulomb repulsion *U* > 0 on the Green function is included in the interaction self-energy matrix 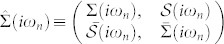, so that the full propagator in the spin-symmetric situation is determined by the Dyson equation 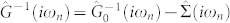. The symmetry relations for the Green function equation (4) reformulated in the Matsubara representation as 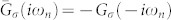 and 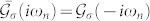 imply the same for the self-energies, i.e. 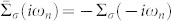 and 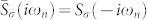. Therefore, the Green function explicitly reads
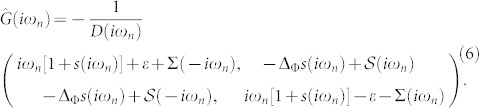


The negative determinant of the inverse Green function 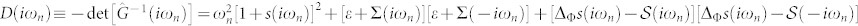 determines via its zeros the existence and positions of the ABS. This determinant is real within the gap and can go through zero *D*(*ω*_0_) = 0 determining the (real) in-gap energies ±*ω*_0_ of the ABS symmetrically placed around the Fermi energy (center of the gap). The ABS are important for transport of the Cooper pairs through the quantum dot and usually provide the dominant contribution to the dissipation-less Josephson current *J* through the impurity, which can be evaluated at zero temperature by an integral of the anomalous Green function (see the Methods section)
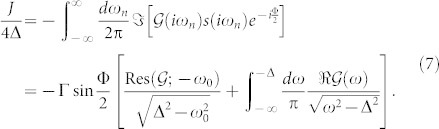
While the first line uses the thermal representation via Matsubara frequencies the second one is the analytic continuation to the real frequencies (spectral representation) which allows us to distinguish the direct supercurrent through the lower ABS (corresponding to the residue of the anomalous impurity Green function at the negative ABS frequency) from the tunneling current between the continuum band states below the SC gap.

### Spin-symmetric Hartee-Fock approximation

As the exact expression for this model's self-energy is unknown we resort to the standard Matsubara perturbation theory summing one-particle irreducible diagrams for the self-energy.

**Figure 1:**
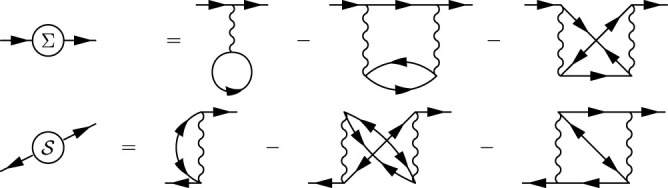
Diagrammatic representation of the first two orders of the perturbation expansion in the Coulomb interaction for the normal (top) and anomalous (bottom) parts of the self-energy. The wavy line represents the Coulomb interaction and the lines with single (double) arrow represent the normal (anomalous) propagators according to equation (4).

The simplest diagrams are the first-order Hartree-Fock contributions represented by the first diagrams on the r.h.s. of equations in Fig. 1. Their mathematical equivalents read
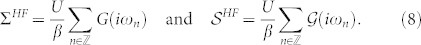


The HF approximation leads just to a static, frequency-independent mean-field self-energy neglecting any dynamical correlations caused by particle interaction. Despite of this simplicity and contrary to the common belief, this approximation yields *without any symmetry breaking* the 0 − *π* quantum phase transition and we thus use it as a convenient and sufficiently simple demonstration of the generic features of the perturbation expansion. The Hartree-Fock approximation consists of two self-consistent non-linear equations that can be reformulated in terms of auxiliary quantities 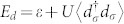 (mean-field energy of the level) and *δ* ≡ Γ cos(Φ/2) + Δ*_d_* (related to the locally induced gap Δ*_d_* ≡ −*U* 〈*d*_↓_*d*_↑_〉). They read
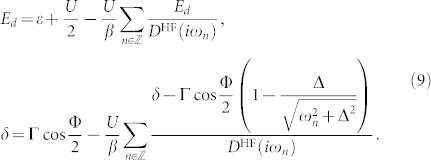
Since we are primarily interested in the zero-temperature QPT where the energies of the ABS approach zero *ω*_0_ → 0, we can approximate the denominators in the integrals by their low-frequency asymptotics 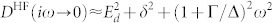, which implies 
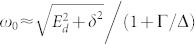
. Near the quantum critical point we then obtain
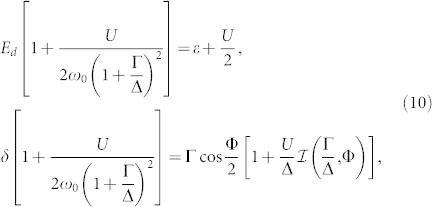
with the band contribution expressed via the function 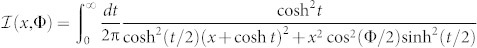. Re-parametrizing *E_d_* = (1 + Γ/Δ)*ω*_0_ cos *ψ* and *δ* = (1 + Γ/Δ)*ω*_0_ sin *ψ* we arrive at
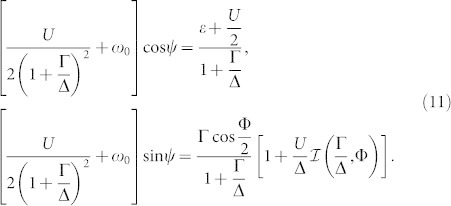


**Figure 2:**
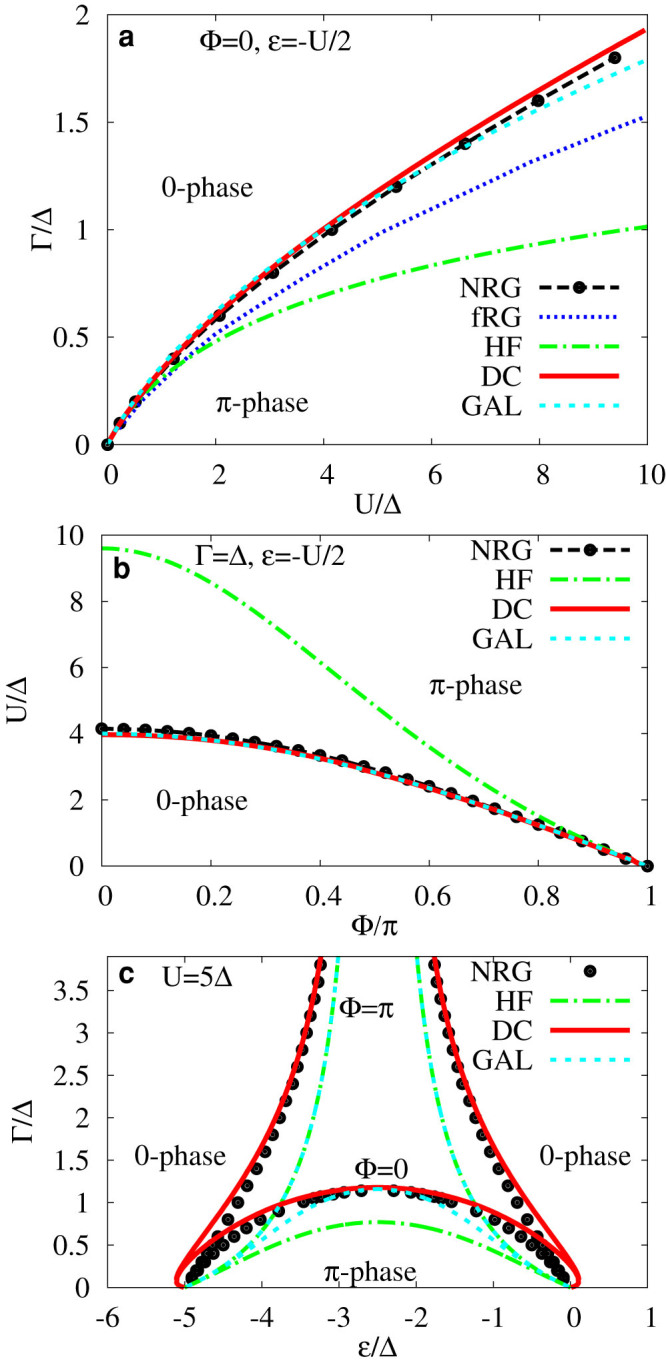
Phase diagrams in the Γ − *U* (a), *U* − Φ (b), and 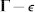 (c) parameter planes. We compare the phase boundaries calculated by numerically exact NRG with various analytical approximations: fRG (only in panel (a); data taken graphically from Fig. 2 of Ref. 27), spin-symmetric HF, the second-order PT/dynamical corrections (DC), and generalized atomic limit approximation (GAL) *U*^2^/(1 + Γ/Δ)^2^ = (2*ε* + *U*)^2^ + 4Γ^2^ cos^2^(Φ/2).

At the QPT characterized by *ω*_0_ = 0 the solubility condition (cos^2^ *ψ* + sin^2^ *ψ* = 1) gives us the equation for the HF phase boundary
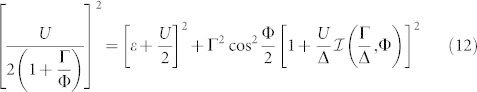
that generalizes the corresponding well-known expression in the atomic limit Δ → ∞^27^,^30^. This HF phase boundary plotted in Fig. 2 is not particularly precise, however, it yields qualitatively reasonable results. Moreover, we have noticed that when the band contribution 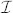 in equation (12) is omitted one gets a surprisingly good and extremely simple approximation for the boundary, that we call here the *generalized atomic limit* (GAL), lying for half-filling (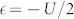) typically very close to the numerically exact results by NRG, see Fig. 2a–b. Obviously, the HF approximation heavily overestimates the contribution from the band continuum.

Eqs. (11) may be used also around the QPT, when *ω*_0_ is small (and unknown). We can see that *ω*_0_ is positive on one side of the boundary while it is negative on the other side. Since *ω*_0_ > 0 by construction, we must conclude that the solution with negative *ω*_0_, that we identify with the *π*-phase region, is unphysical. We cannot go beyond the phase boundary from the 0-phase to the *π*-phase within this perturbative approach based on the assumption of a nondegenerate ground state.

### Dynamical corrections

The qualitative predictions of the HF approximation can be significantly improved by including dynamical corrections into the self-energy, which come from the second order of the perturbation expansion represented by the second and third diagrams on the r.h.s. of equations in Fig. 1. The two diagrams originate in two different types of intermediate propagation consisting of either normal or anomalous propagator. The mathematical equivalents for the second-order contributions read
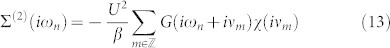
and
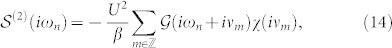
where
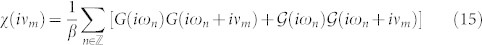
is the two-particle bubble consisting of the normal and anomalous parts and *ν_m_* = 2*πm*/*β* is the *m*-th bosonic Matsubara frequency.

These first two orders of the perturbation expansion are well controllable on the one-particle level. The higher contributions to the self-energy become more complex and their classification more complicated. For a general discussion of this problem see Ref. 31.

The second order self-energy correction together with the first-order (in *U*) HF counterparts are inserted into the equation for the Green function, equation (6). We obtain a self-consistent nonlinear functional equation for the whole Green function as a function of frequency. This equation is solved numerically at zero temperature. We noticed, however, that nearly identical results are obtained by computationally less elaborate method which evaluates the dynamical self-energies by using just a fully converged HF solution as the input GF. The convolutions in the second-order self-energies are thus evaluated just once at the beginning of the procedure and consequently used as fixed inputs into the self-consistent procedure iterating the Green function through the HF self-energy. It should be stressed that while the second-order contribution may be simplified in this way, the full self-consistency loop between the GF and the HF self-energy is mandatory — any compromises there lead to even qualitatively wrong results.

## Discussion

**Figure 3:**
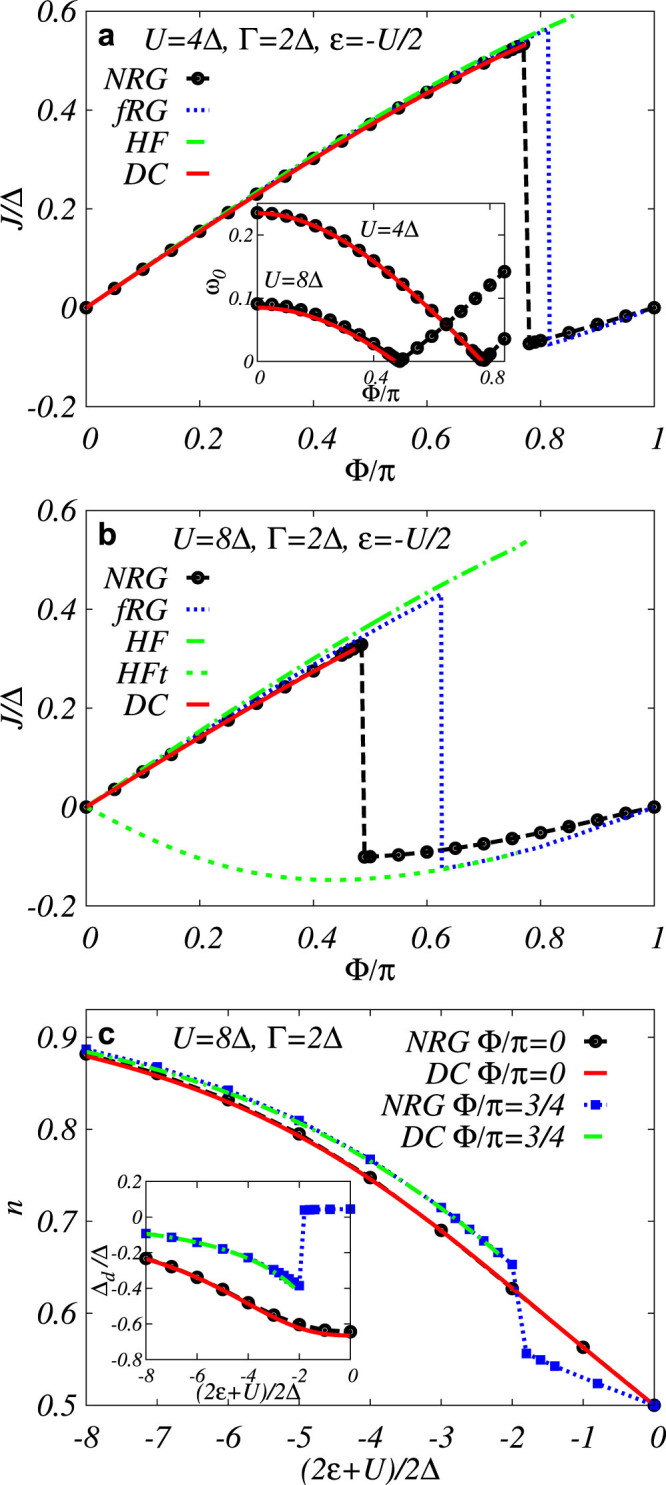
Comparing various methods of calculation of one-particle quantities. Panels (a) and (b) show supercurrent at half-filling as a function of the phase difference Φ for *U* = 4Δ (a) and *U* = 8Δ (b) calculated by numerically exact NRG, and analytically approximative fRG, spin-symmetric HF and, finally, the second-order PT/dynamical corrections (DC) showing a nearly perfect agreement with NRG (unlike the other two methods). Inset in panel (a) depicts the ABS energies *ω*_0_ as functions of Φ for the two values of the Coulomb interaction *U*. The green dashed line in panel (b) represents the HF tunneling current component. In panel (c) the occupation number 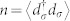 and locally induced SC gap Δ*_d_* ≡ −*U* 〈*d*_↓_*d*_↑_〉 (inset) are plotted as functions of the level energy for two values of the phase difference Φ = 0 (with no phase transition) and Φ = *π* (exhibiting phase transition). fRG data in panels (a), (b) were graphically taken from Fig. 4b of Ref. 27.

We have carried out the above mentioned procedure both in the Matsubara formalism as well as in the spectral representation (performing the analytic continuation described in Methods) with identical results. We have found that the 0-phase smoothly develops from the noninteracting limit *U* = 0 and terminates at the 0 − *π* phase boundary beyond which there exists no regular self-consistent solution for the GF. In the spectral representation this is associated with the energy of ABS *ω*_0_ reaching zero. The results for the phase boundaries, shown in Fig. 2, and one-particle quantities in the 0-phase in Fig. 3 exhibit unprecedented precision of the dynamical corrections approximation which gives numerical results nearly identical to the numerically exact NRG data produced by the “NRG Ljubljana” open source code^32^,^33^ for all studied parameter sets as well as all physical quantities. Surprisingly, it outperforms in the regime of not-so-weak interaction even the fRG method designed for the strong correlations (see the *U*-axis scale in Fig. 2a). This is likely due to the static-vertex implementation of the fRG in Ref. 27. The limitations of the static-vertex approximation have been discussed before (see Ref. 34, Sec. 9.4.6), nevertheless it is currently the only one technically viable for fRG. On the other hand our dynamical corrections properly include the frequency dependence of the correlation effects (even if just perturbatively) which probably explains their superiority over the fRG in the description of 0-phase quantities as well as the phase boundary. In this context, we would also like to point out an interesting observation we have made. In Fig. 3b we plot (by the green dashed line) the tunnelling part of the supercurrent (the second term in the lower equation (7)) for the HF solution and see that it coincides in the overlapping range of parameters with the full supercurrent solution of the fRG in the *π*-phase. Although plotted for clarity just in Fig. 3b this observation holds for all *J* − Φ characteristics taken graphically from Ref. 27. Since our HF solution breaks down at the phase boundary we cannot extrapolate beyond it, nevertheless there is obviously some subtle correspondence between the spin-symmetric HF solution and the *π*-phase solution of the fRG.

To conclude, we have presented a systematic perturbative expansion for the 0 − *π* transition in the superconducting Anderson model and found out that its second order yields at zero temperature excellent results for the phase boundary and quantities in the 0-phase such as locally induced superconducting gap or supercurrent surpassing any (semi)analytical methods employed to this model so far. Although demonstrated here explicitly just for the symmetric case Γ*_L_* = Γ*_R_* for simplicity, the method produces equally good results also in the general case. Moreover, we have also verified numerically that the formalism is gauge-invariant, i.e., physical quantities depend on the phase difference Φ*_L_* − Φ*_R_* only and conserves current, i.e., supercurrents calculated at left/right junctions are identical. Furthermore, the full second-order PT is thermodynamically consistent (unlike, e.g., fRG^34^).

The method cannot be, however, continued to the *π*-phase without modifications taking into account the degeneracy of the doublet ground state. Moreover, we have observed that the Matsubara formalism at finite temperatures does not detect any sharp phase boundary found at *T* = 0. To our best knowledge there is presently no (semi)analytical method that would conceptually correctly and quantitatively reasonably describe the *π*-phase. The spin-polarized HF suffers from the discontinuity problems mentioned in the Introduction while the fRG solution returns *ε*- and *U*-independent quantities in the *π*-phase^27^ apparently closely related with the simplest spin-symmetric Hartree-Fock approximation as discussed above, which is clearly not sufficient. The construction of an analytical theory of the *π*-phase thus remains an open challenge for future study.

## Methods

The necessary information for the study of the crossing of ABS as well as for obtaining the particular components of total current can not be obtained directly from the expressions in Matsubara frequencies. To access it we analytically continued the expressions to the real-frequency domain.

**Figure 4:**
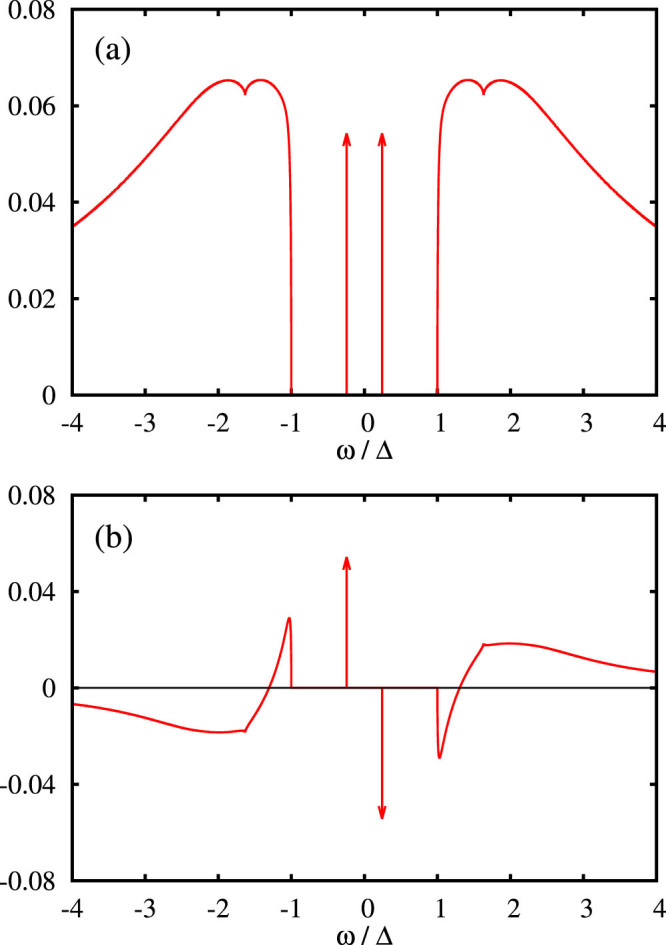
Normal (−Im *G*/*π*, upper panel (a)) and anomalous (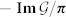, lower panel (b)) spectral density for *U* = 4Δ, Γ = 2Δ, Φ = *π*/2 and *ε* = −*U*/2 (half-filling) calculated using the dynamical corrections from the second-order of the perturbation expansion. The heights of the arrows marking the Andreev bound states represent their residues.

The inverse Green function (4) can be represented as
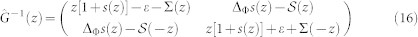
where
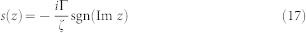
is a dynamical renormalization of the impurity energy level due to the hybridization to the superconducting leads. We introduced a renormalized complex energy *ζ* = *ξ* + *iη* related to *z* = *ω* + *iy* via *ζ*^2^ = *z*^2^ − Δ^2^. The following convention for complex square root is used:
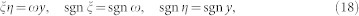
so that *ζ* = *z* for Δ = 0. The renormalized energy *ζ* along the real axis is then real outside the energy gap and imaginary within it. Accordingly to this definition the function *s*(*z*) is imaginary outside the energy gap and real within it,
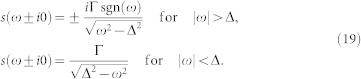
This definition allows for a straightforward analytic continuation of the Matsubara Green function to real frequencies. An illustrating example of the normal and anomalous spectral functions is plotted in Fig. 4. The Green function has a gap around the Fermi energy from −Δ to Δ and two poles at ±*ω*_0_, |*ω*_0_| < Δ. The positions of these poles are given by zeroes of the determinant, 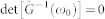. Since the function *s*(*ω*) has a square-root singularity at gap edges, the gap is fixed and does not depend on interaction strength.

Calculating the self-energy from diagrammatic expansion calls for the analytic continuation of sums over Matsubara frequencies. The sum of a one-particle function *F* over fermionic Matsubara frequencies can be rewritten in the spectral representation as^35^
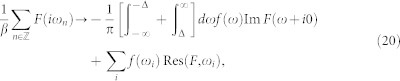
where *ω_i_* are the isolated poles within the gap and *f*(*ω*) is the Fermi-Dirac function. This formula can be used directly to calculate the static Hartree-Fock self-energies (8) and the Josephson current (7).

Similar approach can be utilized to calculate the two-particle bubbles and the second-order dynamic corrections, Eqs. (13)-(15). For the sake of simplicity we resort to zero temperature. Choosing a correct contour in the upper complex half-plane we arrive at an expression for the normal part of the bubble,
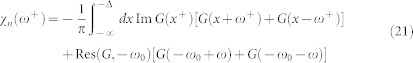
and analogously for the anomalous part *χ_a_*. We have abbreviated *ω*^+^ = *ω*+*i*0. The resulting bubble has an extended gap from −Δ − *ω*_0_ to Δ + *ω*_0_. The contributions from the isolated states at ±2*ω*_0_ from the normal and anomalous parts exactly cancel out each other, so there are no gap states in the full bubble *χ* = *χ_n_* + *χ_a_*. Taking this into consideration we arrive at a formula for the normal self-energy,
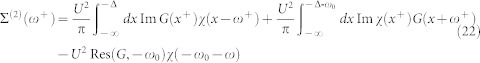
and similarly for 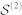. Integrals of this kind can be evaluated numerically using fast Fourier transform algorithms which makes the calculation simple and efficient.

## Author Contributions

M.Ž., V.P., V.J. and T.N. contributed equally to this work.
